# Supplementary material for: Comparative Analysis of Milk Microbiomes and Their Association with Bovine Mastitis in Two Farms in Central Russia
Source: Animals (Basel). 2021 May 14;11(5):1401. doi: 10.3390/ani11051401 (PMC8156869; doi:10.3390/ani11051401)
Supplement: Supplementary file 1 [file animals-11-01401-s001.zip › SuppTable 1.pdf]

Animal	SampleID	SCC ×10^3	Site	Health	Lactation
19	19pp	2868	MR	M	1
3100	3100pp	19	Tula	H	1
5895	5895pp	833	Tula	M	NA
5895	5895zp	453	Tula	S	NA
6494	6494pp	9760	Tula	M	NA
6494	6494zl	40	Tula	H	NA
6539	6539pp	344	Tula	S	NA
6539	6539zp	8539	Tula	M	NA
6730	6730pl	17	Tula	H	1
6814	6814pp	90	Tula	H	1
6814	6814zp	3710	Tula	M	1
6827	6827pl	804	Tula	M	NA
6827	6827pp	54	Tula	H	NA
7012	7012zl	50	Tula	H	NA
7012	7012zp	1165	Tula	M	NA
7021	7021pl	2994	Tula	M	NA
7021	7021pp	219	Tula	S	NA
7029	7029pp	3596	Tula	M	NA
7029	7029zl	153	Tula	S	NA
7040	7040pp	1485	Tula	M	NA
7040	7040zl	156	Tula	S	NA
7197	7197pl	436	MR	S	2
7206	7206zp	10	MR	H	2
7211	7211pl	64	Tula	H	NA
7211	7211pp	1233	Tula	M	NA
7234	7234pp	204	Tula	S	NA
7234	7234zp	4369	Tula	M	NA
7243	7243pl	13	Tula	H	1
7259	7259pl	815	MR	M	2
7259	7259zl	230	MR	S	2
7267	7267pp	4	MR	H	2
7267	7267zl	3	MR	H	2
7276	7276lp	1233	MR	M	2
7276	7276pp	21	MR	H	2
7284	7284lz	2702	MR	M	2
7284	7284pp	245	MR	S	2
7292	7292pl	4	MR	H	2

7309	7309pz	900	MR	M	2
7478	7478lp	272	MR	S	2
7478	7478lz	2023	MR	M	2
7478	7478pp	586	MR	M	2
8510	8510pl	45	Tula	H	NA
8510	8510zl	44	Tula	H	NA
9229	9229zl	5190	MR	M	1
9499	9499pp	651	MR	M	2
9499	9499zl	43	MR	H	2
9697	9697zp	9363	MR	M	1
9711	9711zl	31	MR	H	2
9711	9711zp	735	MR	M	2
9787	9787pp	696	MR	M	1
9787	9787pz	42	MR	H	1
9902	9902lp	114	MR	S	1
9902	9902pp	866	MR	M	1
