# Supplementary figures and images for: Comparative Analysis of Milk Microbiomes and Their Association with Bovine Mastitis in Two Farms in Central Russia
Source: Animals (Basel). 2021 May 14;11(5):1401. doi: 10.3390/ani11051401 (PMC8156869; doi:10.3390/ani11051401)

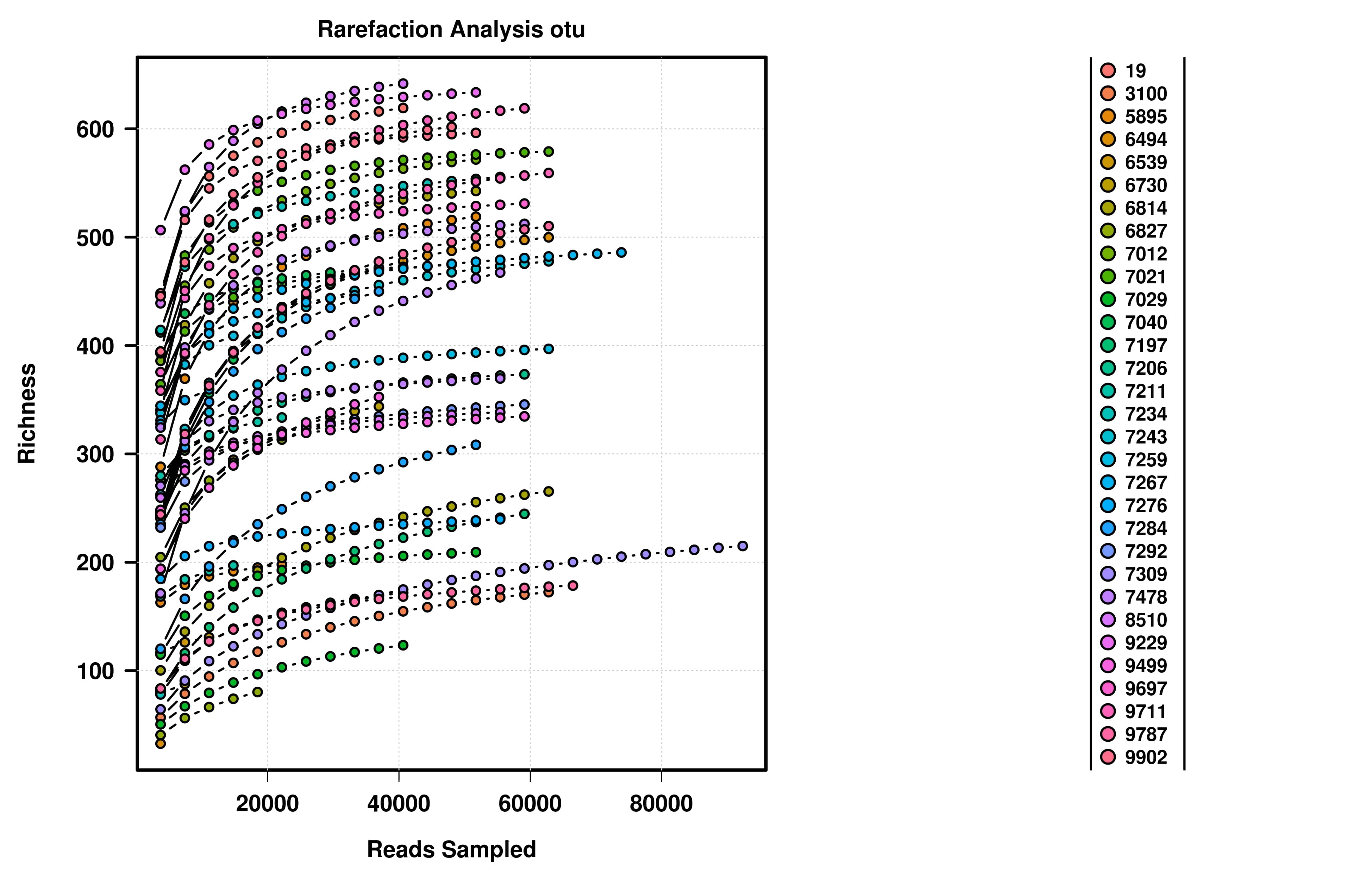

Supplement: Supplementary file 1 [file animals-11-01401-s001.zip › Supplemented material Fig.2.jpg]

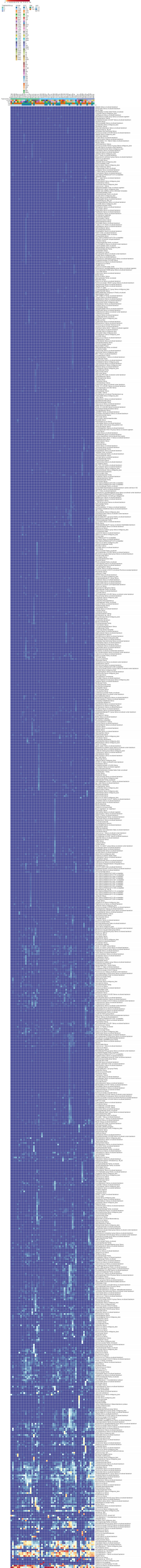

Supplement: Supplementary file 1 [file animals-11-01401-s001.zip › Supplemented material Fig1.jpg]
